# CR6-interacting factor 1 inhibits invasiveness by suppressing TGF-β-mediated epithelial-mesenchymal transition in hepatocellular carcinoma

**DOI:** 10.18632/oncotarget.21925

**Published:** 2017-10-19

**Authors:** Runzhou Zhuang, Di Lu, Jianyong Zhuo, Xuanyu Zhang, Kun Wang, Xuyong Wei, Qiang Wei, Wei Wang, Haiyang Xie, Lin Zhou, Xiao Xu, Shusen Zheng

**Affiliations:** ^1^ Key Lab of Combined Multi-Organ Transplantation, Ministry of Public Health, Hangzhou 310003, China; ^2^ Division of Hepatobiliary and Pancreatic Surgery, Department of Surgery, First Affiliated Hospital, Zhejiang University School of Medicine, Hangzhou 310003, China; ^3^ Collaborative Innovation Center for Diagnosis and Treatment of Infectious Diseases, Hangzhou 310003, China

**Keywords:** CR6-interacting factor 1, hepatocellular carcinoma, TGF-β, EMT

## Abstract

CR6-interacting factor 1 (CRIF1) regulates cell cycle progression and the DNA damage response. Here, we show that CRIF1 expression is decreased in hepatocellular carcinoma (HCC) tissues and positively correlates with patients’ survival. *In vitro*, down-regulation of CRIF1 promotes HCC cell proliferation and invasiveness, while over-expression has the opposite effect. *in vivo*, CRIF1 knockdown enhances growth of HCC xenografts. Analysis of mRNA microarrays showed that CRIF1 knockdown activates genes involved in TGF-β RI/Smad2/3 signaling, leading to epithelial-mesenchymal transition (EMT) and increased matrix metalloproteinase-3 (MMP3) expression. However, cell invasion and EMT are abrogated in HCC cells treated with SB525334, a specific TGF-β RI inhibitor, which indicates the inhibitory effect of CRIF1 on HCC tumor growth is mediated by TGF-β signaling. These results demonstrate that CRIF1 benefits patient survival by inhibiting HCC cell invasiveness through suppression of TGF-β-mediated EMT and MMP3 expression. This suggests CRIF1 may serve as a novel target for inhibiting HCC metastasis.

## INTRODUCTION

Deaths resulting from hepatocellular carcinoma (HCC) rank third in all malignancy-related mortality [1]. In China, liver cancer is the most commonly diagnosed cancer and the leading cause of cancer death in men under the age of 60 years [2]. Surgical resection and liver transplantation are the best options for HCC treatment. However, majority of HCC patients are not eligible for surgical operation due to metastasis, which is also a major obstacle to cure.

Systemic dissection of the molecular mechanisms underlying metastatic progression of HCC is necessary for the development of new diagnostic and therapeutic strategies to prevent and treat metastases. Epithelial-to-mesenchymal transition (EMT) is a key event in metastasis [3, 4]. CR6-interacting factor 1 (CRIF1), also known as Gadd45gip1 or PRG6, regulates cell cycle by inhibiting G1 to S phase progression, and binds to Gadd45 family [5], which plays an important role in genomic stability and regulation of the cell cycle. However, the precise role of CRIF1 in HCC has not been investigated.

In this study, we evaluated the expression of CRIF1 in HCC tissues, and investigated its function in regulating HCC cell proliferation, *in vivo* tumor growth, EMT, and HCC metastasis.

## RESULTS

### CRIF1 expression is decreased in HCC patients, and correlates with patients’ survival

**Figure 1:**
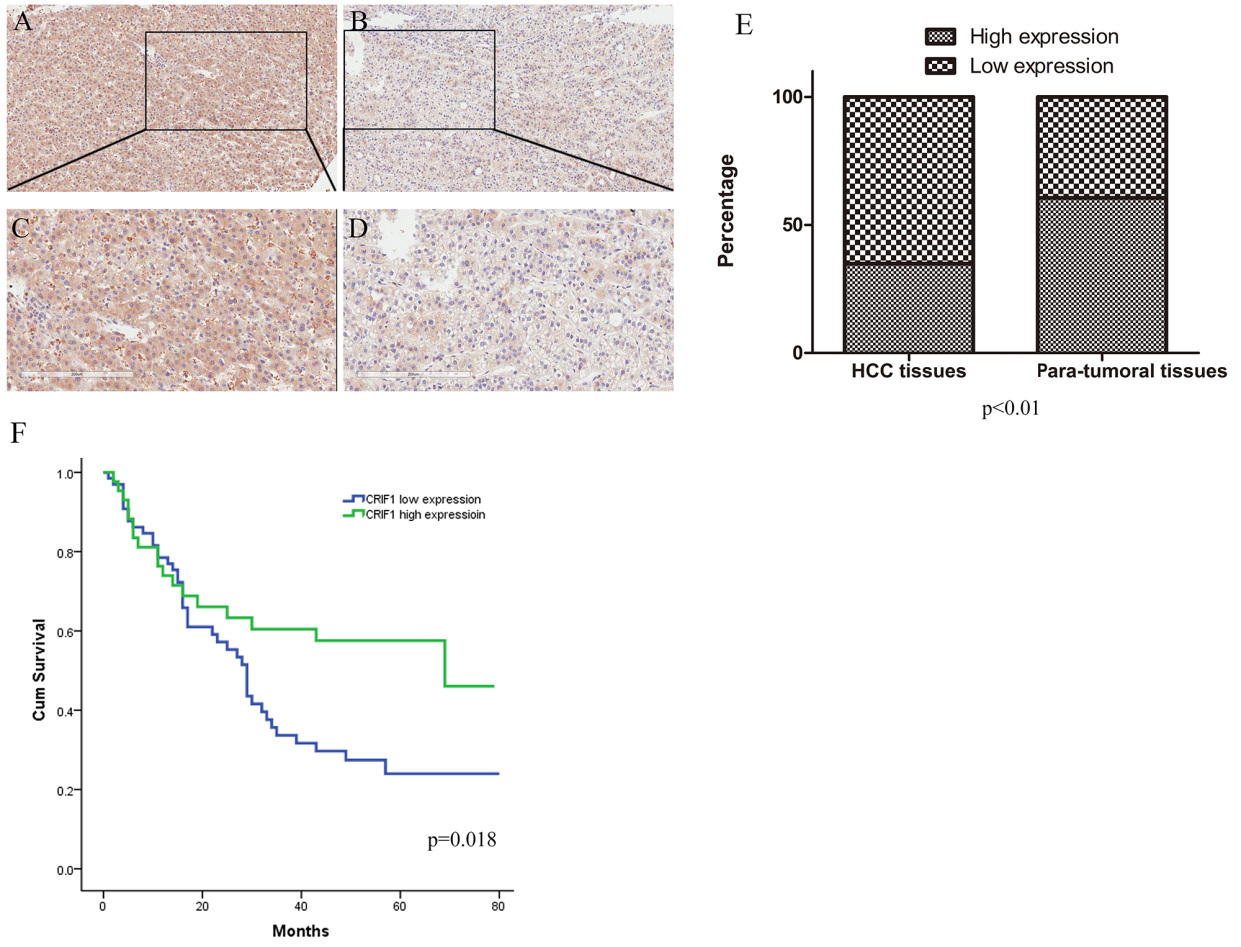
Immunohistochemical staining and prognosis value of CRIF1 Immunohistochemical analysis showed that the CRIF1 protein expression was lower in HCC tissues **(B and D)** than in paracarcinoma tissues **(A and C)**. (A, B: 100× magnification; C, D: with 200× magnification). **(E)** CRIF1 was downregulated in the tumor tissues (P<0.01). **(F)** Its expression level was negatively correlated with the prognosis of HCC patients (P=0.018).

First, we analyzed CRIF1 expression in human HCC tissues by immunohistochemistry (IHC). 38 out of 109 HCC tissues (34.9%) had high CRIF1 levels (staining score>6), while 66 of the 109 para-tumor tissues (60.6%) had high CRIF1 levels (chi-square test, P<0.001). CRIF1 was downregulated in HCC tissue (Figure 1A, 1B, 1C, 1D and 1E). Pearson correlation analysis revealed that the CRIF1 protein level was tightly related to tumor differentiation (P = 0.049) and diameter (P = 0.047) (Table 1). Univariate analysis demonstrated that tumor diameter, differentiation, and CRIF1 expression were of statistical significance. As shown in Figure 1F, Decreased CRIF1 expression in HCC tissues was associated with poor post-operative survival (P=0.018). The overall three-year survival rates for the low CRIF1 and high CRIF1 groups were 25.8% and 48.8% respectively (P=0.023).

**Table 1:** Associations between CRIF1 protein expression level and clinic-pathological features of 109 patients with hepatocellular carcinoma

Variables	score		p
	0-6	7-12	
Age (≤50, >50)	17/49	13/30	0.609
Tumor diameter (≤5, >5cm)	22/44	23/20	0.047
Cirrhosis (with/without)	40/26	24/19	0.619
Differentiation (I/II/III)	1/30/35	2/25/16	0.049
AJCC stage (1, 2/3,4)	8/21/33/4	10/17/14/2	0.228

### CRIF1 suppression promotes proliferation of HCC Huh-7 and SK-HEP-1 cells

**Figure 2:**
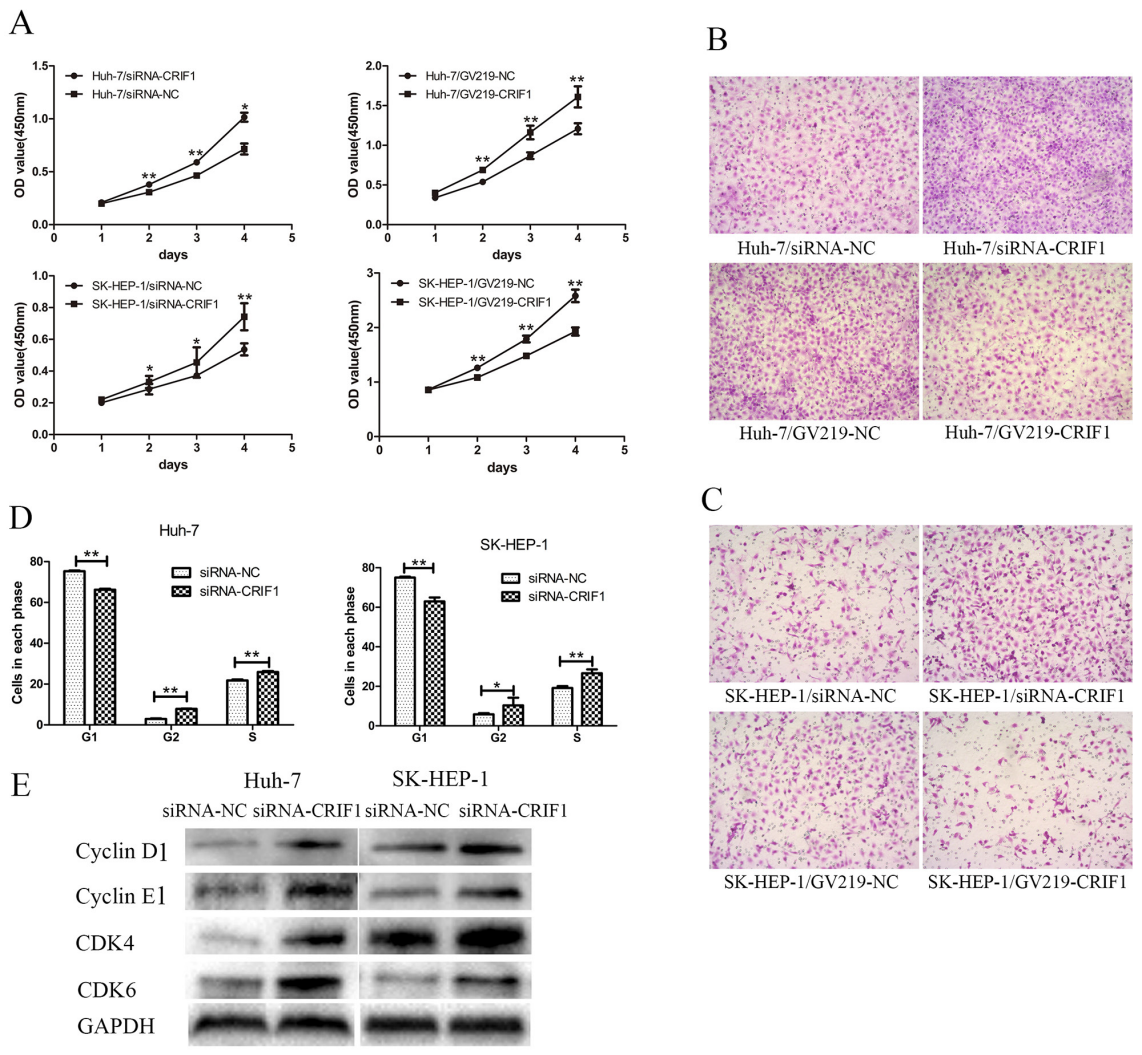
The effects of CRIF1 on phenotype of HCC cell lines **(A)** Knockdown of CRIF1 promoted cell proliferation, while enforced expression of CRIF1 inhibited it in Huh-7 and SK-HEP-1. **(B, C)** The invasive potential of HCC cells was increased in the siRNA-CRIF1 group, and decreased in the GV219-CRIF1 group. **(D)** CRIF1 knockdown decreased the number of G1 phase cells and increased the number of G2 and S phase cells in hepatocellular carcinoma cell lines. **(E)** CRIF1 knockdown resulted in elevation of Cyclin D, Cyclin E, CDK4 and CDK6. (^**^: P<0.01, ^*^:0.01<p<0.05).

CRIF1 knockdown promoted cell proliferation and invasive potency, while restoration of CRIF1 had the opposite effect (Figures 2A, 2B and 2C). Flow cytometry revealed that CRIF1 knockdown decreased the number of G1 phase cells and increased the number of G2 and S phase cells (Figure 2D). In accordance, proteins controlling G1/S transition, including cyclin D1, cyclin E1, CDK4, and CDK6 were significantly upregulated after CRIF1 knockdown (Figure 2E).

### CRIF1 inhibits HCC tumorigenicity in mice

**Figure 3:**
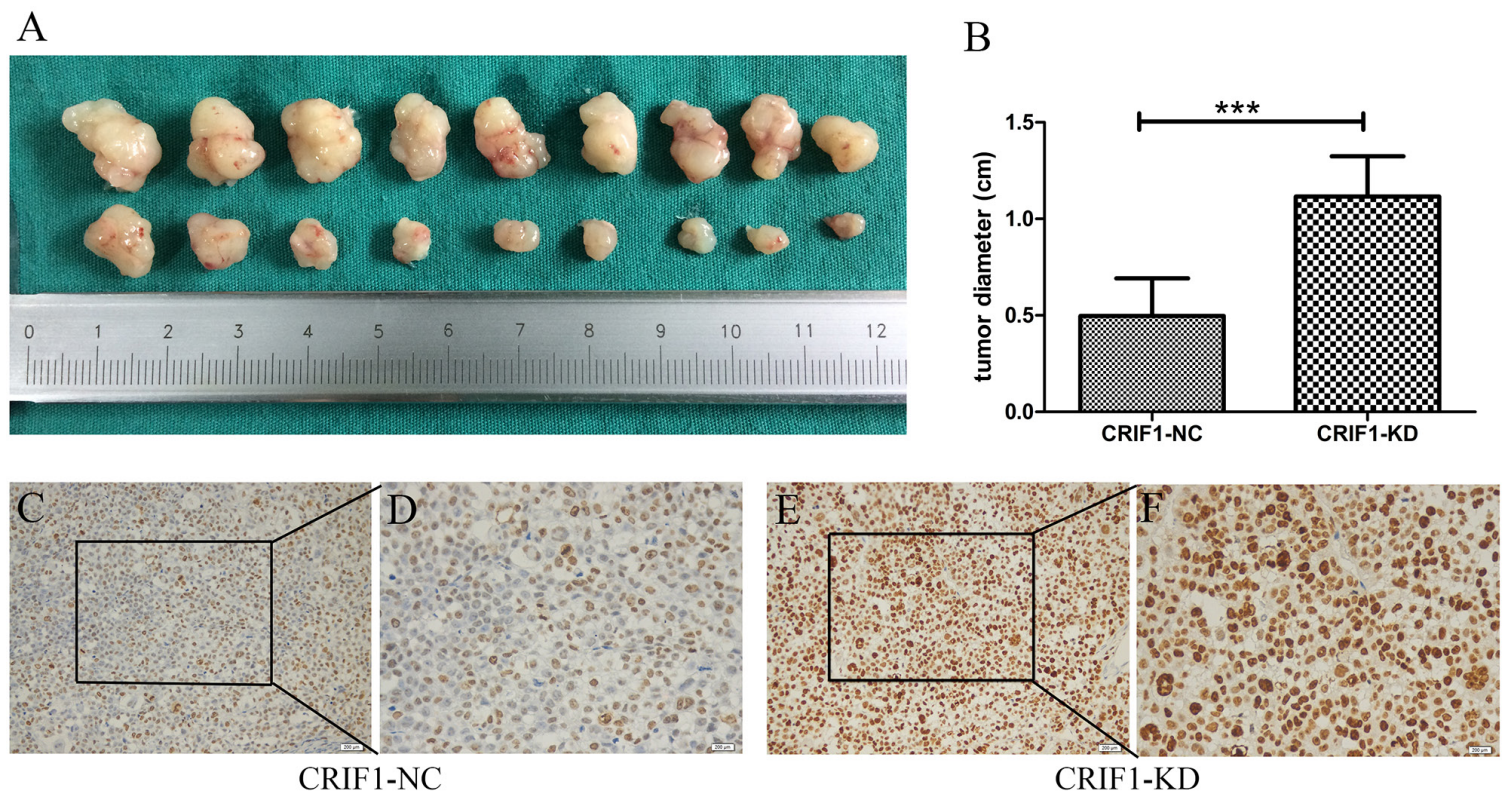
**(A, B)** Effect of stable CRIF1 on xenograft tumor size 4 weeks after injection of HCC cells. IHC staining of Ki67 for the xenograft tumor of the lenti-CRIF group **(C, D)** and lenti-NC group **(E, F)**; 200× and 400 ×, magnification).

To evaluate the role of CRIF1 in promoting HCC tumorigenicity *in vivo*, mice were subcutaneously injected with HCC cells with CRIF1 knockdown (CRIF1-KD) or normal control (NC) cells. As shown in Figure 3, four weeks after injection, the CRIF1-KD group had significantly larger tumors (1.115 +/- 0.209 cm, n=8) than the CRIF1-NC group (0.496 +/- 0.196 cm, n=8, P<0.001). Xenograft tumors in the CRIF1-KD group had elevated percentage of Ki-67 positive cells compared to the control group (Figures 3C–3F).

### Gene expression microarray

**Figure 4:**
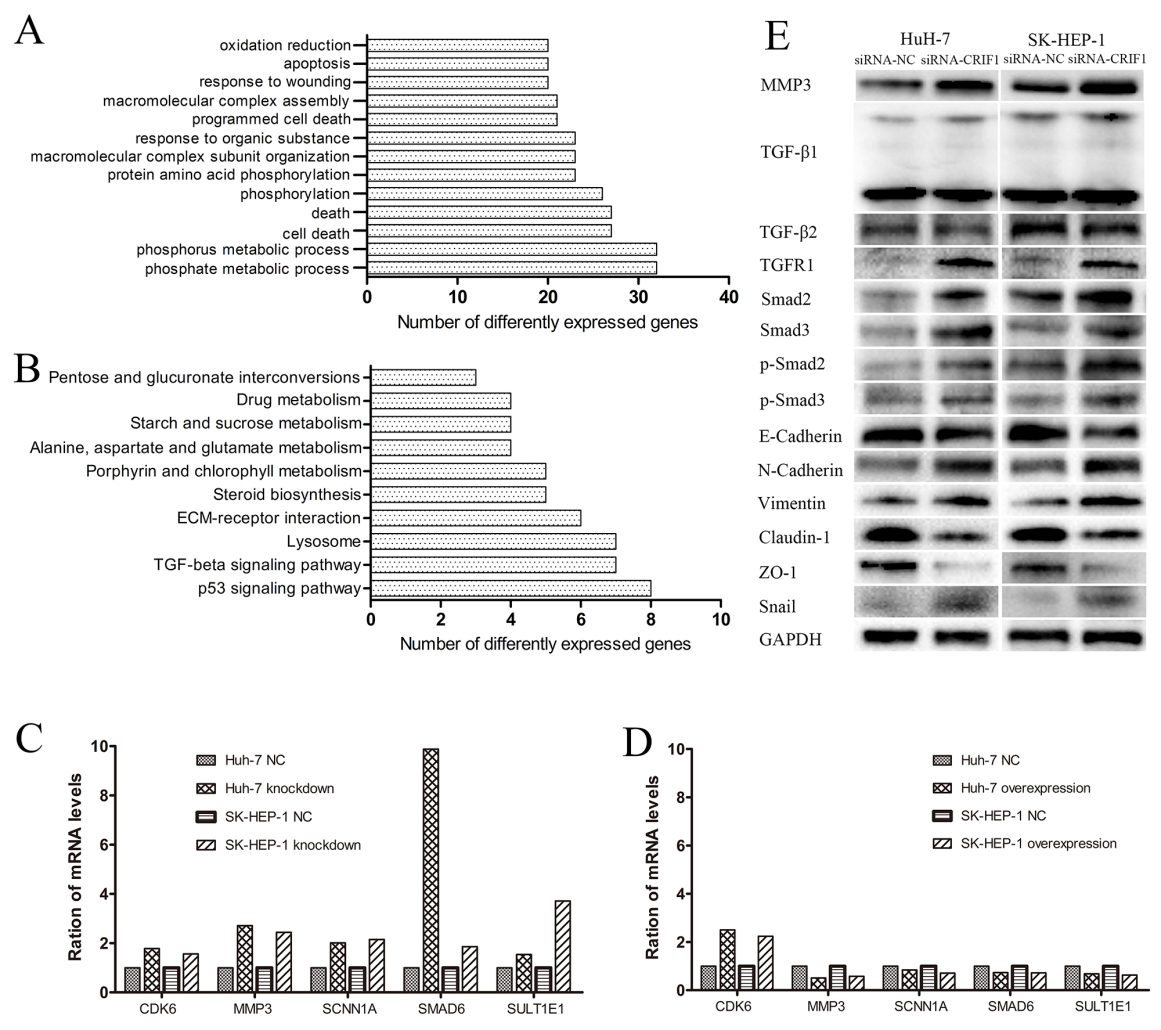
Analysis and validation of the mRNA microarray **(A and B)** The gene ontology and KEGG analyses of differentially expressed genes identified by microarray. **(C, D)** Validation of the most significantly altered genes in the array by RT-PCR. **(E)** Validation of the TGF-β signaling and EMT-associated proteins by western blot.

A total of 527 differentially expressed genes (fold change>2, p < 0.05) were identified between SK-HEP-1 cells transfected with siRNA-CRIF1 or siRNA-NC. The data were further analyzed with an online database (http://david.abcc.ncifcrf.gov/). The gene ontology analysis (Figure 4A) and Kyoto Encyclopedia of Genes and Genomes (KEGG) analysis (Figure 4B) indicated involvement of genes and signaling pathways involved in the regulation of cell death, apoptosis, adhesion, and migration. Among them, MMP3 exhibited the most significant change. Knockdown of CRIF1 in HCC cells increased the mRNA expression of MMP3, Smad6, CDK6, and several other genes, while restoration of CRIF1 decreased the expression of these genes (Figures 4C and 4D).

### TGF-β signaling is activated by CRIF1 knockdown in HCC cells

In KEGG analysis, TGF-β signaling was increased following CRIF1 knockdown. In accordance, we found increased expression of TGF-β receptor I, Smad2, Smad3, phosphorylated Smad2 (p-Smad2) and phosphorylated Smad3 (p-Smad3) (Figure 4E), indicating activation of TGF-β/Smad signaling in CRIF1 suppressed HCC cells.

**Figure 5:**
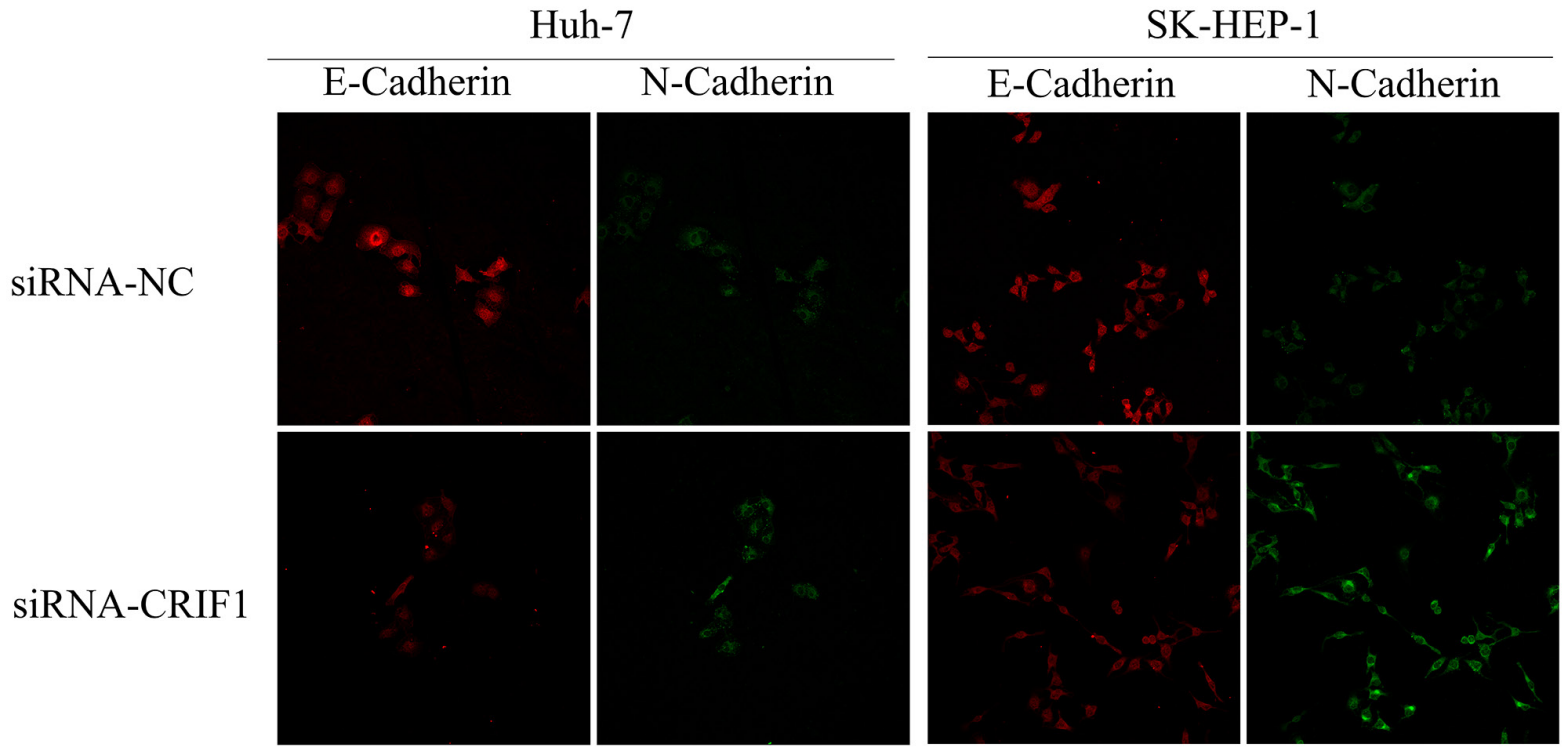
Immunofluorescent double staining of E-Cadherin and N-Cadherin

**Figure 6:**
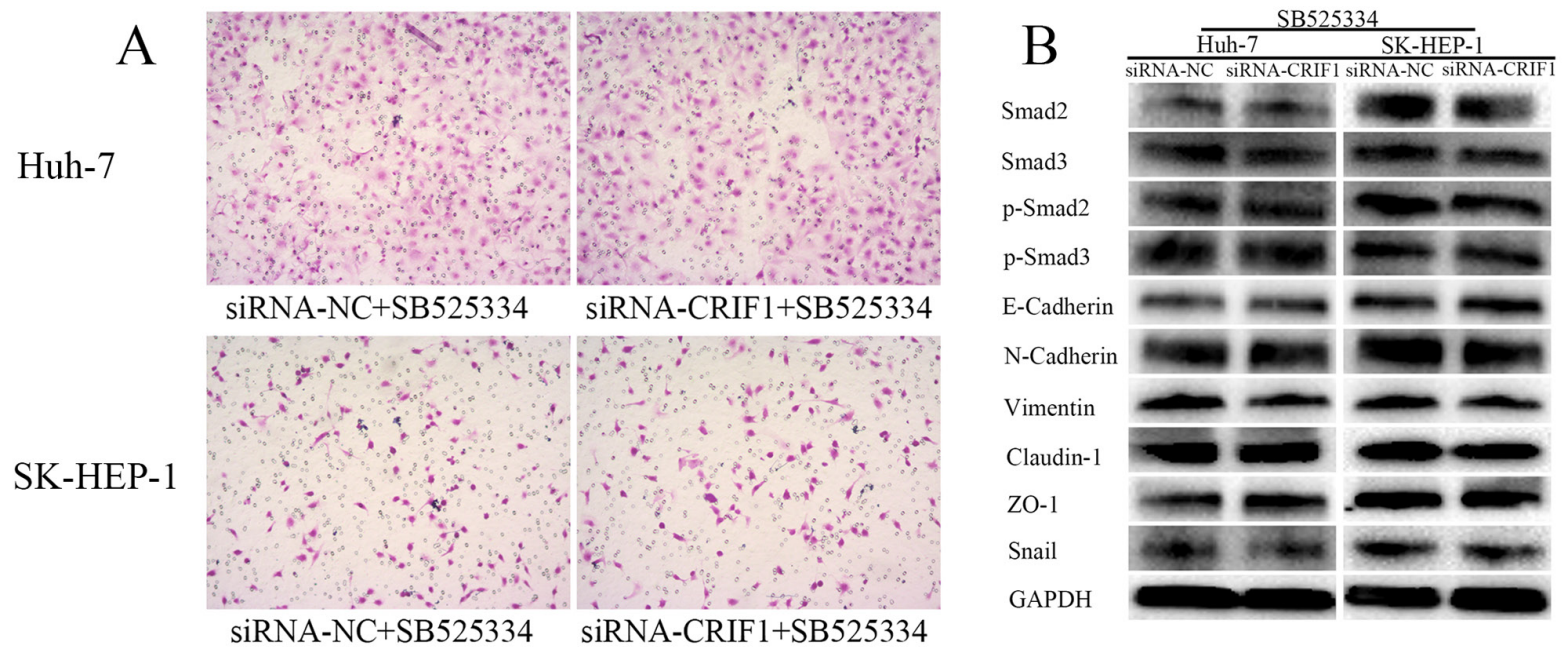
The effects of CRIF1 knockdown was abrogated by TGF-β receptor I specific inhibitor SB525334 **(A)** Increase in invasive potency of CRIF1 knockdown was abrogated by SB525334. **(B)** Alteration of TGF-β signaling and EMT-associated proteins were abrogated by SB525334.

Since the activation of TGF-β signaling causes EMT and tumor metastasis, we investigated the impact of CRIF1 suppression on EMT in HCC cells. As shown in Figure 4E, we found a decrease of epithelial marker E-cadherin and elevation of mesenchymal markers N-cadherin and vimentin in CRIF1-suppresed cells. Snail, which binds to E-cadherin promoter region to repress transcription, was also upregulated. Meanwhile, tight junction-associated proteins claudin and ZO-1 were downregulated. Double immunofluorescent staining of E-cadherin and N-cadherin confirmed that CRIF1 knockdown promoted EMT (Figure 5). When treated with TGF-β receptor I specific inhibitor SB525334, CRIF1 knockdown-activated TGF-β signaling, invasion, and EMT were abrogated (Figure 6), suggesting that CRIF1 suppression promotes invasion and EMT in Huh-7 and SK-HEP-1 cells through TGF-β/Smad signaling.

## DISCUSSION

Metastases account for the great majority of cancer-associated deaths, yet this complex process remains the least understood aspect of cancer biology [6]. Unraveling the mechanism of metastasis will open a new avenue to cancer cure. CRIF1 inhibits cell cycle, and functions as an essential STAT3 coactivator [7]. CRIF1 induces cell cycle arrest in the G1 phase by inhibiting the kinase activity of Cdk2-cyclin complexes, Cdk1-cyclin B1and histone H1 [8, 9]. However, the role of CRIF1 in HCC has not been investigated.

Our data indicate that CRIF1 inhibits HCC cell proliferation, decreases the percentage of G1 phase cells, and inhibits *in vivo* tumorigenicity of HCC xenografts. In addition, our results are the first to demonstrate that CRIF1 inhibits HCC cell invasiveness, since CRIF1 knockdown increased HCC cells invasive potency, while restoring CRIF1 expression decreased their invasive potency.

Microarray data have indicated that several important genes including MMP3, PTEN, Smad6, and CDK6 are regulated by CRIF1 [10–12]. Among them, MMP3, a member of the matrix metalloproteinase (MMP) family, which is involved in the breakdown of extracellular matrix in processes, such as tissue remodeling and metastasis [13], had the largest fold change. The observed changes in MMP3 protein levels following CRIF1 knockdown or overexpression, may represent one of the mechanisms of how CRIF1 regulates invasiveness of HCC cells.

TGF-β signaling, which plays pivotal roles in tumor metastasis [14], was among the most altered signaling pathways following CRIF1 knockdown. CRIF knockdown increased Smad2, Smad3, p-Smad2 and p-Smad3 expression, suggesting activation of the TGF-β signaling. Expression of TGF-β receptor I was increased after CRIF1 knockdown, implying that TGF-β receptor I plays a driving role in CRIF1-regulated TGF-β signaling.

TGF-β1 has a differential regulatory effect on MMPs. It up-regulates MMP2 and MMP9, down-regulates MMP1 and MMP8 [15–18], and has no effect on MMP3 [19]. In our study, we found that blocking TGF-β signaling did not affect MMP3 expression in HCC cells. TGF-β signaling and MMP3 are involved in EMT [20–24]. MMP3 directly cleaves the extracellular domain of E-cadherin, prompting normal mammary epithelial cells to disaggregate and undergo EMT [25]. Pharmacological inhibition of MMP3 abrogates the TGF-β1-triggered cell invasiveness [26]. In this study, we found elevation of MMP3 and activation of TGF-β signaling following CRIF1 knockdown, so we sought to investigate the role of EMT in CRIF1 regulated cell invasiveness. As expected, CRIF1 knockdown decreased E-cadherin, and increased N-cadherin.

To confirm our hypothesis that CRIF1 regulates HCC EMT through TGF-β signaling, we treated CRIF1-KD and CRIF1-NC cells with SB525334, a TGF-β receptor I (ALK5) specific inhibitor [27–29]. Since blocking the TGF-β receptor inhibited the HCC cell invasiveness and EMT, these data indicate that CRIF1 regulates cell invasion via TGF-β-activated EMT and MMP3 expression. EMT and MMP3 have been considered as candidate targets for cancer therapy [30–35]. For example, galunisertib, TGF-β receptor I inhibitor, has been associated with strong improvement of HCC in the phase II trial [36].

Together, our results reveal a novel function of CRIF1 in regulating EMT and MMP3 expression in HCC cells, and suggest that CRIF1 may be an ideal target for inhibiting HCC metastasis.

## MATERIALS AND METHODS

### Clinical sample preparation

This study was approved by the Ethical Committee of the First Affiliated Hospital of Zhejiang University School of Medicine, and conducted in accordance with the ethical principles of the Declaration of Helsinki. All clinical samples used in this study were obtained from patients after informed consents. A total of 109 paired specimens of HCC were randomly enrolled in this study from HCC patients undergoing hepatectomy from 2010 to 2012 at the First Affiliated Hospital of Zhejiang University School of Medicine.

### Reagents and cell culture

The human HCC cell lines Huh-7 and SK-HEP-1 were purchased from Chinese Academy of Sciences Shanghai cell bank and cultured in complete growth medium, as recommended by the manufacturer. Cells were maintained at 37°C in a humidified incubator with 5% CO2. The TGF-β RI specific inhibitor SB525334 was purchased from Sellleck (Selleck Chemicals, Houston, TX, USA), and used at a concentration of 1 μM.

### Quantitative real-time PCR (qRT-PCR)

Total RNA was extracted from tissues or cells with Trizol reagent (Invitrogen, USA). qRT-PCR was carried out using the master SYBR Green kit (Takara Bio, Japan) according to the manufacturer’s instruction by ABI Prism 7500 fast sequence detection system (applied Biosystem, USA). All reactions were performed in triplicates, and analyzed by using the 2^-∆∆^Ct method. The results were normalized to mRNA expression of GAPDH, which was used as internal control.

### Western blotting

Total proteins were extracted from tissues and cells. Anti-GAPDH antibody (1:5000, A5441, Sigma), anti-CRIF1 (1:2000, 16260-1-AP, Proteintech), anti-cyclin D1 (1:2000, ab137875, Abcam), anti-cyclin E1 (1:1000, ab52189, Abcam), anti-CDK4 (1:1000, ab137675, Abcam), anti-CDK6 (1:1000, ab151247, Abcam), anti-MMP3 (1:1000, ab52915, Abcam), anti-TGF-β1 (1:500, ab9758, Abcam), anti-TGF-β2 (1:100, ab167655, Abcam), TGF-β receptor 1 (1:1000, ab155258, Abcam), anti-Smad2 (1:1000, 3122, CST), anti-Smad3 (1:1000, 9513, CST), anti-p-Smad2 (1:1000, 3108, CST), anti-p-Smad3 (1:1000, 9520, CST), anti-E-Cadherin (1:2000, ab133597, Abcam), and anti-N-Cadherin (1:1000, ab19348, Abcam) antibodies were used. Immunodetection was performed using an EZ-ECL chemiluminescence detection kit (BeitHaemek, Israel).

### Immunohistochemistry

Immunohistochemistry (IHC) was performed on HCC tissues using anti-CRIF1 (1:100, ab111254, Abcam) and anti-Ki67 (1:50, ab833, Abcam) antibodies. Immunostaining of CRIF1 was semi-quantitatively scored by multiplying the values of the mean staining intensity and the percentage of CRIF1-positive cells. The intensity of staining was scored as absent (0), weakly positive (1), moderately positive (2), or strongly positive (3). The percentage of CRIF1-positive cells was scored as no cells (0), less than 10% of cells (1), 11–50% of cells (2), 51–80% of cells (3), and more than 80% of cells (4). Finally, cases were grouped as low expression (scores 0–6) or high expression (scores 7–12).

### CRIF1 siRNA interference, and recombinant plasmid and lentivirus infection

siRNA-CRIF1 and siRNA-NC (negative control) were synthesized by Invitrogen. The siRNA sequence for CRIF1 was ACACCUAGUAGGCUGCGUGCCUGUC. Hu-7 and SK-Hep-1cells were cultured in 6-well plates in MEM. When cells were about 30% confluent, they were transfected with siRNA- CRIF1 or siRNA-NC using transfection reagent (Lipofectamine 2000, Invitrogen, Carlsbad, CA). The CRIF1-recombinant and NC vectors were constructed by GeneChem. Stable CRIF1 knockdown (CRIF1-KD) and its negative control (CRIF1-NC) cell lines were obtained by infection of lentivirus targeting CRIF1 and the negative control. The efficiency of transfections was validated by qRT-PCR and western blotting. Fluorescence of CRIF1-KD and CRIF1-NC cells was observed after transfection and puromycin screening (Supplementary Figure 1).

### Cell proliferation assay

Cell proliferation was measured using the Cell Counting Kit-8 (Dojindo, Japan) assay. Briefly, pretreated HCC cells (transfected with siRNA or GV219) were placed in 96-well plates (normally 4000 cells per well) and cultured for indicated times. The absorbance was measured at 450 nm using Absorbance Reader (BIO-TEK ELX800); the experiments were repeated 3 times.

### Tumor invasion assay

Polycarbonate membranes Transwell (Corning, USA) and Matrigel (BD, USA) were used for the migration assay. After different treatments, a total of 5×10^4^ Hu-7 and SK-Hep-1cells were suspended in 200 μL of serum-free medium and seeded into the upper chamber, while 600 μL of MEM containing 10% FBS was added to the lower chamber. After incubation at 37°C, the cells on the upper membrane were removed with cotton swabs. The membranes were fixed in paraformaldehyde solution and stained with 0.5% crystal violet. Cells on the lower surface of the membrane were counted in randomly selected fields with microscope (100×). The experiment was independently repeated three times.

### Xenograft tumorigenicity assay

All animal experiments were performed according to the Zhejiang University Animal Care Facility and National Institutes of Health guidelines.

Six-week-old BALB/c male nude mice (average weight 21 g) were randomly divided into two groups (8 mice each). Pretreated SK-Hep-1cells (4×10^6^ cells in 0.1ml PBS) were injected subcutaneously into the nude mice. Tumor volumes were measured after mice were sacrificed.

### Cell cycle and apoptosis assays

For cell cycle analysis, cells were harvested and fixed in ice-cold 75% ethyl alcohol at 4° C overnight. After incubation with a DNA PREP kit solution (Beckman Coulter, Fullerton, CA) in the dark for 30 min, cell apoptosis was detected by FACS. Data were analyzed using ModFit LT3.1 software. Apoptosis was detected in HCC cells not expressing GFP (Green Fluorescent Protein) using an FITC Annexin V Apoptosis Detection Kit II (BD, San Jose, CA), and in cells expressing GFP using an Annexin V Apoptosis Detection Kit APC (eBioscience, SanDiego, CA). Cells were analyzed using a flow cytometer (LSR II; BD Bioscience). All experiments were conducted in triplicates.

### Statistical analysis

All values are expressed as mean ± standard deviation. Student’s t-test was applied to evaluate statistical significance. The variable was converted to a logarithm if it did not conform to a normal distribution. Χ^2^ test was used to detect the difference between categorical variables. The endpoint for follow-up was patient death. Overall survival rates were calculated using the Kaplan–Meier method. Log-rank tests were performed for univariate analysis. P-values less than 0.05 were considered statistically significant. All statistical analyses were performed using SPSS 11.0software (SPSS, Chicago, IL).

## SUPPLEMENTARY MATERIALS FIGURE



## Abbreviations

CRIF1: CR6-interacting factor 1
HCC: hepatocellular carcinoma
EMT: Epithelial-to-mesenchymal transition
MMP: matrix metalloproteinase
