# Supplementary material for: CR6-interacting factor 1 inhibits invasiveness by suppressing TGF-β-mediated epithelial-mesenchymal transition in hepatocellular carcinoma
Source: Oncotarget. 2017 Oct 19;8(55):94759–68. doi: 10.18632/oncotarget.21925 (PMC5706910; doi:10.18632/oncotarget.21925)
Supplement: Supplementary file 1 [file oncotarget-08-94759-s001.pdf]

CR6-interacting factor 1 inhibits invasiveness by suppressing TGF-β-mediated epithelial-mesenchymal transition in hepatocellular carcinoma

SUPPLEMENTARY MATERIALS

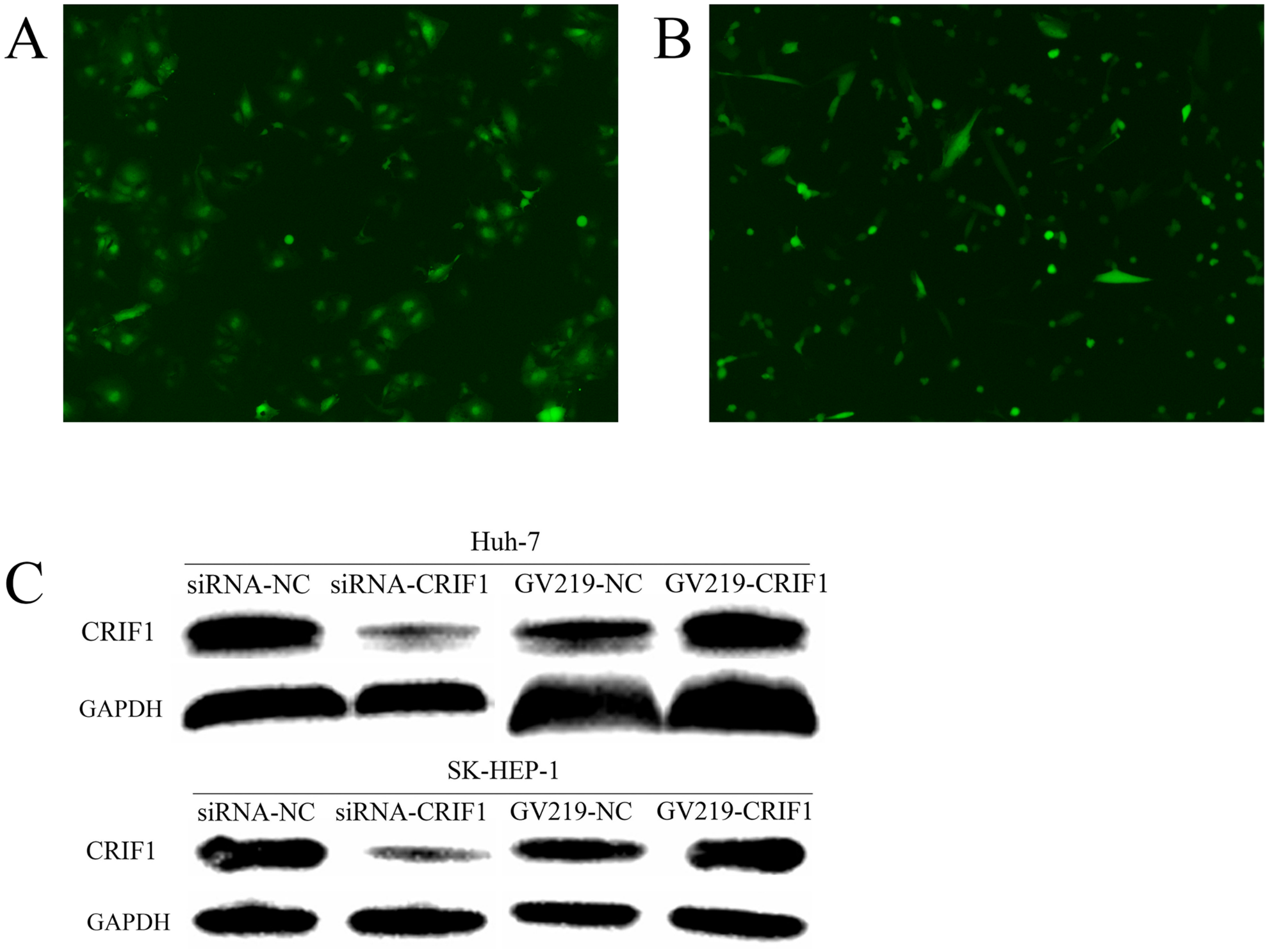

Supplementary Figure 1: siRNA and lentivirus efficiency validation. (A, B). Image of Huh-7 and SK-HEP-1 cells after GFP-labeled lentivirus infection. **(C)**. CRIF1 expression was significantly altered after transfection.
